# Health Care Practitioner Bias and Access to Inpatient Rehabilitation Services Among Survivors of Violence

**DOI:** 10.1001/jamanetworkopen.2025.4074

**Published:** 2025-04-08

**Authors:** Megan R. Georges, Alexa Courtepatte, Alice Hibara, Jennifer Harris, Tanesha Beckford, David Wiley, Emma Weinberger, Rebecca Rudel, Elizabeth Dugan, Jonathan Jay, Elizabeth C. Pino

**Affiliations:** 1 Violence Intervention Advocacy Program, Department of Emergency Medicine, Boston Medical Center, Boston, Massachusetts; 2 Department of Community Health Sciences, Boston University School of Public Health, Boston, Massachusetts; 3 Department of Emergency Medicine, Brigham and Women’s Hospital, Massachusetts General Brigham, Boston; 4 Division of Violence Prevention, Boston Public Health Commission, Boston, Massachusetts; 5 Section of Infectious Diseases, Department of Medicine, Boston University Chobanian & Avedisian School of Medicine, Boston, Massachusetts; 6 Evans Center for Implementation and Improvement Sciences, Department of Medicine, Boston University Chobanian & Avedisian School of Medicine, Boston, Massachusetts; 7 Department of Emergency Medicine, Boston Medical Center, Boston, Massachusetts; 8 Department of Emergency Medicine, Boston University Chobanian & Avedisian School of Medicine, Boston, Massachusetts

## Abstract

**Importance:**

Posthospital inpatient rehabilitation is essential for many patients with traumatic injuries. However, rehabilitation centers lack transparency and oversight in their admission practices and may be influenced by health care practitioner (HCP) use of stigmatizing language in patient medical records, leading to inequities in access to care.

**Objectives:**

To examine differences in admission to inpatient rehabilitation centers for patients hospitalized for violent penetrating (VP) injuries compared with motor vehicle crash (MVC) injuries.

**Design, Setting, and Participants:**

This mixed-methods retrospective qualitative study used data obtained from hospital records from 2015 to 2021. Data analysis occurred between July and December 2023. The study was performed at Boston Medical Center, an urban level I trauma center. The cohort included all patients hospitalized for VP or MVC injuries who were discharged to an inpatient rehabilitation center between 2015 and 2021.

**Exposures:**

Injury type, categorized as VP or MVC.

**Main Outcomes and Measures:**

The primary quantitative outcome was a denial for admission to an inpatient rehabilitation center. Qualitative content analysis identified similarities and differences across injury types in the manifestations of predetermined stigmatizing language categories in patients’ medical records.

**Results:**

Of 323 patients discharged to an inpatient rehabilitation center (median [IQR] age, 38 [25-59] years; 208 men [64.4%]; 29 Hispanic patients [9.0%], 118 non-Hispanic Black patients [36.5%], and 152 non-Hispanic White patients [47.1%]), 107 patients (33.1%) experienced at least 1 denial by a center before being placed (32 of 55 patients with VP injuries [58.2%] vs 75 of 268 patients with MVC injuries [28.0%]). Compared with patients with MVC injuries, patients with VP injuries had greater than 3 times the odds of experiencing a denial (odds ratio, 3.51; 95% CI, 1.93-6.48; *P* < .001). Medical records of patients with VP injuries had increased use of stigmatizing language that indicated culturally based or injury-related stereotyping, skepticism toward patient-reported symptoms, and heightened HCP-power dynamics contributing to unilateral decision-making.

**Conclusions and Relevance:**

In this mixed-methods qualitative study of hospital patients discharged to rehabilitation centers, significant disparities in denials for admission were observed among survivors of violence, who were disproportionally Black or Hispanic. Stigmatizing language found in medical records suggested that bias within the referral process may have contributed to these disparities. These findings underscore the need for reformed clinical documentation practices and enhanced oversight of rehabilitation referral processes to promote equitable access to care.

## Key Points

**Question:**

Are hospitalized patients with violent injuries more likely to be denied admission to posthospital inpatient rehabilitation?

**Findings:**

In this qualitative study that included 323 patients who were discharged to inpatient rehabilitation with either violent penetrating or motor vehicle crash injuries, patients with violent injuries had more than 3 times higher odds of being denied admission by rehabilitation centers. Patients with violent penetrating injuries had distinct instances of stigmatizing language associated with injury-specific biases in their medical records.

**Meaning:**

These findings highlight the need to address barriers to care for survivors of violence and the role that biased language in medical records can play in perpetuating health disparities.

## Teaser

This qualitative study examines differences in admission to inpatient rehabilitation centers for patients hospitalized for violent penetrating injuries compared with patients with motor vehicle crash injuries as well as use of stigmatizing language in patient health records.

## Introduction

Patients with traumatic injuries often require posthospital inpatient rehabilitation services to recover from severe injuries.^1^ Nearly 25% of patients with traumatic injuries are ultimately discharged from the hospital to inpatient rehabilitation centers, including inpatient rehabilitation facilities, long-term acute care hospitals, and skilled nursing facilities.^2^ Rehabilitation improves patient functional status and independence during a particularly vulnerable time for survivors of traumatic injuries,^3^ increasing patient quality of life after injury.^4,5^ Appropriate rehabilitation following hospitalization is considered a critical juncture in the continuum of care for patients with traumatic injury, and interruptions or delays in postdischarge care contribute to disparities in adverse health outcomes,^6^ including higher risks for preventable rehospitalization and 1-year mortality, as well as decreased engagement in follow-up care.^3,5^

The rehabilitation referral process is coordinated by hospital case managers (CMs) and based on the medical care team’s recommendations according to a patient’s functional status, ongoing medical needs, willingness to participate in rehabilitation, home environment, and anticipated recovery progress.^7,8^ If inpatient rehabilitation placement is needed, CMs identify appropriate options for the patient’s required level of care, insurance, and geographic preferences. Referral packets include care recommendations and pertinent segments from a patient’s medical record.^9^ Facilities determine acceptance or denial of a patient based on their independent evaluation of the patient’s status and internal policies.^8^ Hospital health care practitioners (HCPs), insurance companies, and rehabilitation centers often disagree over the most appropriate course of posthospital care for patients.^8,10^ If denied by a rehabilitation center, CMs continue the referral process until a patient is placed or their needs change.

Previous work suggests that acceptance criteria are inconsistently applied, with nonclinical factors and industry regulations affecting placement.^8,10,11,12^ Prior studies have revealed disparities in access to rehabilitation centers by race, ethnicity, insurance coverage, and referring hospital.^6,10,13,14,15^ Black and Hispanic patients are less likely to be discharged to a rehabilitation center than non-Hispanic White patients, as are uninsured compared with insured patients with traumatic injuries,^15,16^ potentially contributing to higher rates of mortality.^17^ Survivors of violence are another population that may experience disparate access, especially young males of color, such as Black and Hispanic men, who report being treated like gang members or criminals in clinical settings.^18,19,20^ This discrimination reinforces racial inequities in health care, since survivors of violence are disproportionately Black and Hispanic youth,^21^ and hampers the trust needed to deliver essential services, eg, hospital-based violence intervention, designed to interrupt cycles of violence and socioeconomic instability that often affect survivors of violence.^22,23^ Additionally, pervasive stigmatizing language in medical records (eg, expressions of disapproval, discrediting, stereotyping, portraying the patient as difficult, unilateral decision-making^24^) incorporates bias that can impact clinical decision-making, potentially perpetuating health disparities and influencing the rehabilitation placement process.^25,26,27^

The objectives of this mixed-methods study were to evaluate possible disparities in patient denials to rehabilitation centers among survivors of violence and explore the role of HCPs’ use of stigmatizing language in patient medical records. Survivors of violence are discharged from the hospital to their home at higher rates than patients with nonviolent trauma injuries.^28^ However, to our knowledge, this is the first analysis to assess inequities in denials to rehabilitation centers for patients with trauma injuries using both qualitative and quantitative methods. We hypothesized that survivors of violence would have higher odds of experiencing a rehabilitation center denial and more instances of stigmatizing language written in their medical records. This project was motivated by conversations with hospital-based violence intervention workers at Boston Medical Center (BMC), whose experience providing direct services to survivors of violence helped generate the study hypotheses.

## Methods

This qualitative study was approved by the Boston University, BMC institutional review board and determined to be not greater than minimal risk (under 45 CFR 46). This study was granted waivers of Health Insurance Portability and Accountability Act (HIPAA) authorization (under 45 CFR164.512 [i] [2] [ii]) and informed consent (under 45 CFR 46.116(f)).

The Boston University, BMC institutional review board approved this study with a waiver of Health Insurance Portability and Accountability Act (HIPAA) of 1996 authorization for research and deemed this study exempt from federal regulations for the protection of human research participants. Quantitative results were reported following the Strengthening the Reporting of Observational Studies in Epidemiology (STROBE) reporting guideline, and qualitative results were reported following the Standards for Reporting Qualitative Research (SRQR) reporting guideline. Data analysis occurred in July through December 2023.

### Study Design and Population

This mixed-methods retrospective qualitative study included patients hospitalized at BMC for initial encounters for a traumatic injury between 2015 and 2021 who discharged to a rehabilitation center, including inpatient rehabilitation facility, long-term acute care hospital, or skilled nursing facility. Survivors of violence were those hospitalized for a violent penetrating (VP) injury (ie, gunshot or stab wound). We compared these individuals with patients hospitalized for nonviolent injuries, specifically from motor vehicle crashes (MVCs). We drew the study sample only from individuals who ultimately discharged to rehabilitation centers to ensure they had confirmed clinical needs for rehabilitation, allowing us to examine the experiences of patients with VP injuries within the rehabilitation placement process. Patients across injury types were seeking placement within the same group of facilities, with 81% of the population ultimately discharging to 1 of 10 facilities. The BMC Clinical Data Warehouse for Research supported the data strategy and provided data extracts for this research. Patient race and ethnicity were self-reported and categorized as Hispanic, non-Hispanic Black, non-Hispanic White, and other or unknown (including American Indian or Alaska Native and Asian individuals, as well as those who chose not to report their race or ethnicity).

Quantitative analysis was performed on the entire cohort of patients. A subset of patients was selected for deductive qualitative content analysis^29^ of all notes in their medical records from their hospital admission, which are found in BMC’s electronic health records. A flowchart of inclusion criteria is presented in the eFigure in Supplement 1. We selected a convergent parallel mixed-methods approach, which allowed us to gain a more comprehensive understanding of the rehabilitation referral process through independent analysis of the 2 datasets.^30,31^

### Quantitative Measures and Analysis

The primary exposure of interest was patient injury type: MVC compared with VP injuries. The primary quantitative outcome was a patient receiving at least 1 denial for admission to a rehabilitation center, assessed as a dichotomized variable. Our secondary outcomes were the number of rehabilitation denials per patient and the associated reasons, which were primarily documented by CMs and manually abstracted from health record notes for the encounter. Additional quantitative measures and analysis details are described in eAppendix 1 in Supplement 1.

### Qualitative Data Collection and Analysis

A subset of patient records (25 patients with VP injuries; 25 patients with MVC injuries) were identified through random sampling stratified by injury type using a random number generator. Random sampling was applied disproportionately to strata to obtain a sufficient sample for the underrepresented violent injury type.^32^ We analyzed the text of all health record notes (eg, notes written by physicians, physician’s assistants, nurse practitioners, nurses, CMs, physical therapists, occupational therapists, and social workers) for each patient’s entire hospital admission for the injury of interest. Based on current literature^25,26,33,34^ and discussions with frontline violence intervention program personnel^35^ and an emergency medicine physician, we developed a codebook anchored in the 5 stigmatizing language categories and definitions identified by Park et al^24^ from their review of physician use of stigmatizing language in medical charts: (1) questioning credibility, (2) difficult patient, (3) disapproval, (4) stereotyping, and (5) unilateral decisions. Additional details of the qualitative analysis are described in eAppendix 2 in Supplement 1.^25,29,33,36,37^

### Statistical Analysis

Categorical variables were compared using χ^2^ test or Fisher exact test. Continuous variables were compared using the Wilcoxon rank sum test. We used logistic regression to estimate crude odds ratios (ORs) and ORs adjusted for injury type and whether hospital public safety was called. *P* values were 2-sided, and statistical significance was set at *P* ≤ .05. Data were analyzed using R software version 4.3.2 (R Project for Statistical Computing).

## Results

### Quantitative

There were 323 patients (median [IQR] age, 38 [25-59] years; 208 men [64.4%]; 29 Hispanic patients (any race) [9.0%], 118 non-Hispanic Black patients [36.5%], and 152 non-Hispanic White patients [47.1%], 24 patients with other or unknown race or ethnicity [7.4%]) included for analysis who were hospitalized between 2015 and 2021 and discharged to an inpatient rehabilitation center. Overall, 55 patients (17.0%) were treated for VP injuries and 268 patients (83.0%) were treated for MVC injuries. Patient and hospital characteristics are detailed in Table 1. Patients treated for violent injuries were younger, more likely to be male, more likely to identify as Black or Hispanic, and had longer hospital lengths of stay than patients with MVC injuries (Table 1). HCP notes in patient records revealed that patients with VP injuries were more likely to have a housing barrier, meaning they would be without a stable location for discharge following rehabilitation (VP: 7 patients [12.7%]; MVC: 6 patients [2.2%]; *P* = .001), and substantially more likely to have a rehabilitation center require the contact information of law enforcement with jurisdiction over the incident related to the patient’s injury (VP: 40 patients [72.7%]; MVC: 7 patients [2.6%]; *P* < .001).

**Table 1.** Characteristics of Patients With Violent Penetrating and Motor Vehicle Crash Injuries Discharged to Inpatient Rehabilitation Centers, 2015-2021

Characteristic	Patients, No. (%)			*P* value^a^
	All (N = 323)	By injury type		
		Violent penetrating (n = 55 [17.0%])	Motor vehicle crash (n = 268 [83.0%])	
Age, median (IQR), y	38 (25-59)	28 (23.5-39)	42 (26-65)	<.001
Gender				
Female	115 (35.6)	10 (18.2)	105 (39.2)	.005
Male	208 (64.4)	45 (81.8)	163 (60.8)	
Race and ethnicity				
Hispanic (any race)	29 (9.0)	8 (14.5)	21 (7.8)	<.001
Non-Hispanic Black	118 (36.5)	38 (69.1)	80 (29.9)	
Non-Hispanic White	152 (47.1)	2 (3.6)	150 (56.0)	
Other or unknown^b^	24 (7.4)	7 (12.7)	17 (6.3)	
Length of stay, median (IQR), d	10.0 (6.0-18.5)	16.7 (10.1-27.5)	9.0 (6.0-16.0)	<.001
Noted in patient record during hospitalization				
Housing barrier^c^				
Yes	13 (4.0)	7 (12.7)	6 (2.2)	.001
No	310 (96.0)	48 (87.3)	263 (97.8)	
Mental health diagnosis^d^				
Yes	77 (23.8)	15 (27.3)	62 (23.1)	.62
No	246 (76.2)	40 (72.7)	206 (76.9)	
Hospital public safety called^e^				
Yes	19 (5.9)	5 (9.1)	14 (5.2)	.42
No	304 (94.1)	50 (90.9)	254 (94.8)	
Police contact^f^				
Yes	47 (14.6)	40 (72.7)	7 (2.6)	<.001
No	276 (85.4)	15 (27.3)	261 (97.4)	

^a^ Categorical variables were compared using χ^2^ tests. Continuous variables were compared using the Wilcoxon rank sum test.

^b^ Other or unknown race and ethnicity includes patients who were American Indian or Alaskan Native or Asian or who chose not to respond when asked about their race and ethnicity.

^c^ Housing barrier indicates that the patient’s medical record during their hospitalization included an HCP’s note explicitly stating that the patient did not have stable housing to discharge to from a rehabilitation center.

^d^ Mental health diagnoses included those listed in patients’ past medical history or explicitly noted by an HCP in their records: anxiety, depression, attention-deficit/hyperactivity disorder, bipolar disorder, posttraumatic stress disorder, schizophrenia, or borderline personality disorder.

^e^ Hospital public safety called indicates that it was documented in a patient’s chart that public safety was paged by hospital HCPs for assistance with either the patient or associated visitors, including remaining on standby, escorting visitors out of the hospital, and assisting with the application of violent restraints to a patient.

^f^ Police contact indicates that a patient’s record noted that a rehabilitation center required being provided the contact information of law enforcement assigned to any criminal investigation related to the events that resulted in a patient’s injuries to complete admission screening.

Overall, 107 patients (33.1%) were denied admission to a rehabilitation center for any reason (VP: 32 patients [58.2%]; MVC: 75 patients [28.0%]; *P* < .001) (Table 2). Compared with patients with MVC injuries, patients with VP injuries were more likely to be denied explicitly due to safety concerns commonly ascribed to patients with VP injuries (VP: 8 patients [14.5%]; MVC: 0 patients; *P* < .001) and for unspecified reasons (VP: 15 patients [27.3%]; MVC: 27 patients [10.1%]; *P* = .001) and had a higher number of rehabilitation denials (median [IQR], VP: 1 [0-2] denials; MVC: 0 [0-1] denials; *P* < .001). There were no differences by injury type for denials due to insurance coverage, bed availability, or patient level of care. Patients with more denials had longer hospital stays (median [IQR] hospital length of stay, 0 denials, 9.0 [5.5-12.5] days; ≥2 denials, 20.3 [9.9-30.6] days) (eTable 2 in Supplement 1).

**Table 2.** Reasons for Facility Denials for Admission to Inpatient Rehabilitation Centers

Reason	Patients, No. (%)			*P* value^a^
	All (N = 323)	Injury type		
		Violent penetrating (n = 55 [17.0%])	Motor vehicle crash (n =286 [83.0%])	
Denied for admission to inpatient rehabilitation	107 (33.1)	32 (58.2)	75 (28.0)	<.001
Denials, median (IQR) [range]	0 (0-1) [0-8]	1 (0-2) [0-7]	0 (0-1) [0-8]	<.001
Denied due to insurance coverage	30 (9.3)	8 (14.5)	22 (8.2)	.22
Denied due to bed availability at center	24 (7.4)	8 (14.5)	16 (6.0)	.05
Denied due to patient level of care^b^				
Overall	35 (10.8)	9 (16.4)	26 (9.7)	.23
Patient requires more care: discrepancies in recommendations	24 (7.4)	5 (9.1)	19 (7.1)	
Patient requires more care: mental health	5 (1.5)	1 (1.8)	4 (1.5)	
Patient requires more care: substance use	1 (0.3)	0	1 (0.4)	
Patient requires more care: COVID-19 positivity	2 (0.6)	0	2 (0.7)	
Patient requires less care: discrepancies in recommendations	9 (2.8)	2 (3.6)	7 (2.6)	
Denied due to housing concerns for postrehabilitation discharge	4 (1.2)	0	4 (1.5)	>.99
Denied for safety concerns, eg, victim of violence	8 (2.5)	8 (14.5)	0	<.001
Denied for unspecified reasons	42 (13.0)	15 (27.3)	27 (10.1)	.001

^a^ Categorical variables were compared using χ^2^ test or Fisher exact test. Continuous variables were compared using the Wilcoxon rank sum test.

^b^ Denials due to discrepancies in recommended level of care indicates that either the rehabilitation center or patient’s insurer denied admission due to divergent assessments of appropriate level of care recommended by hospital and/or rehabilitation center.

Multivariable models estimated that patients with VP injuries had greater than 3 times the odds of being denied admission to a rehabilitation center compared with patients with MVC injuries (odds ratio [OR], 3.51; 95% CI, 1.93-6.48; *P* < .001) (Table 3; eTable 1 in the Supplement). In our univariate models, Black race, compared with all other race and ethnicity categories, was associated with greater odds of rehabilitation center denial (odds ratio [OR], 2.06; 95% CI, 1.23-3.46; *P* = .006). Despite established disparities in access to rehabilitation services for racial and ethnic minority groups,^10,15,17^ and a greater percentage of Black and Hispanic patients with VP injuries in our study population compared with patients with MVC injuries (89.1% vs 42.6%), race and ethnicity were not significant factors associated with rehabilitation center denials in multivariable analysis, and model comparison using a likelihood ratio test revealed no significant decline in model fit when removing race and ethnicity.

**Table 3.** Patient-Level Factors Associated With Any Facility Denial During the Discharge Process to Inpatient Rehabilitation Centers

Characteristic	Patients denied, No. (%) (n = 107 [33.1%])	Odds of rehabilitation denial^a^			
		Crude		Final multivariable model	
		OR (95% CI)	*P* value	OR (95% CI)	*P* value
Injury type					
Motor vehicle crash	75 (28.0)	1 [Reference]	<.001	1 [Reference]	<.001
Violent penetrating	32 (58.2)	3.58 (1.98-6.58)		3.51 (1.93-6.48)	
Age	NA	0.99 (0.98-1.00)	.23	NA	NA
Injury year	NA	0.98 (0.87-1.09)	.67	NA	NA
Gender					
Female	35 (30.4)	1 [Reference]	.44	NA	NA
Male	72 (34.6)	1.21 (0.75-1.99)		NA	
Race and Ethnicity					
Hispanic (any race)	10 (34.5)	1.52 (0.63-3.51)	.05	NA	NA
Non-Hispanic Black	49 (41.5)	2.06 (1.23-3.46)		NA	
Non-Hispanic White	39 (25.7)	1 [Reference]		NA	
Other or unknown^b^	9 (37.5)	1.74 (0.68-4.23)		NA	
Housing barrier					
No	99 (31.9)	1 [Reference]	.03	NA	NA
Yes	8 (61.5)	3.41 (1.11-11.53)		NA	
Mental health diagnosis					
No	75 (30.5)	1 [Reference]	.08	NA	NA
Yes	32 (41.6)	1.62 (0.95-2.75)		NA	
Hospital public safety called					
No	96 (31.6)	1 [Reference]	.02	1 [Reference]	.04
Yes	11 (57.9)	2.98 (1.17-7.92)		2.81 (1.07-7.66)	
Police contact					
No	79 (28.6)	1 [Reference]	<.001	NA	NA
Yes	28 (59.6)	3.67 (1.95-7.05)		NA	

Abbreviations: NA, not applicable; OR, odds ratio.

^a^ Logistic regression models were used for estimating ORs, 95% CIs and *P* values. The multivariable model is adjusted for injury type and whether hospital public safety was called.

^b^ Other or unknown race and ethnicity includes patients who were American Indian or Alaskan Native or Asian or who chose not to respond when asked about their race and ethnicity.

### Qualitative

Content analysis indicated the presence of each of the 5 stigmatizing language categories in medical records of both injury types (Table 4). We observed that instances of biased language in the categories questioning credibility, stereotyping, and unilateral decisions occurred more frequently in the records of patients with VP injuries. HCP documentation of discharge planning commonly encapsulated the category unilateral decisions, along with injury-specific stereotyping against patients with VP injuries. Notes with language suggesting questioning credibility reflected increased skepticism of patient-reported symptoms for patients with VP injuries. The categories difficult patient and disapproval occurred in similar fashion across injury types.

**Table 4.** Categories of Stigmatizing Language, Adapted Definitions, Examples From Patient Medical Records, and Summaries of Findings

Category	Adapted definition	Examples by injury type		Summary of findings
		VP	MVC	
Questioning credibility	HCPs not believing patients, implying their inability to convey accurate information, implying lack of competency, doubting genuineness of symptoms, and questioning patients’ adherence to treatment	“states he cannot lift his right arm although several times earlier in the day he had his arms above his head and was using them to pull himself up in bed”	“Unrestrained driver who claims being dizzy while driving & hit a car”; “Pt endorses ETOH use but denies prior to accident, despite serum result.”	HCP notes framed patients as unreliable narrators. The words “apparently” and “claims” more commonly prefaced reports for patients with VP injuries of incident details, and HCPs’ selective use of quotations sometimes indicated skepticism. Records of patients with VP injuries also included greater disbelief in patients’ symptom and pain reporting. In notes, HCPs combined patients’ self-reporting with juxtaposed clinical context in attempt to discredit them. Superfluous commentary insinuated patients’ lack of competency and impaired judgment, often overlapping with the disapproval theme
Disapproval	Use of language that targets patients’ reasoning, decision-making, and behavior (including self-care, lifestyle, circumstances surrounding injury, and hospital presentation), at times implying a tiresome need for repetition of information	“Pt stated he uses when he is out with friends because he ‘just wants to’.”; “Pt has poor coping skill. He cries, gets angry, throws thing, then wants hugs. Pt acts childlike at times”	“She states that she has driven after drinking alcohol but reports that she does so ‘safely’ (though also felt that she was ‘safe’ to drive on the night of her accident”	HCPs’ notes included comments scrutinizing patients’ judgment and behavior while receiving medical care, including perceived lack of comprehension, participation, and adherence with care. HCPs sometimes expounded on patients’ social circumstances, incorporating quotations to record a patient’s diction or indicate judgment toward patients’ perspectives. This sometimes extended to patients’ visitors. The most evident instances of HCP disapproval were notes containing transitional or conjunctive adverbs, such as “however,” “unfortunately,” and “despite” and statements ending with ellipses. This theme commonly overlapped with the difficult patient theme, and there were no notable differences in theme representation across injury types.
Stereotyping	Explicit racially charged language, documenting nonclinical information inappropriately or unnecessarily, reflecting a belief about a patient based on a certain characteristic, or use of patients’ quotes that highlight grammar, wording, slang, or use of unsophisticated medical terms	“Pt has a 4 yo dtr [name] who lives with ‘baby mother’”; “[Rehabilitation center name] did get in touch w/ the [city] police detective assigned to pt’s case and unfortunately will not be offering a bed due to safety concerns.”	“He reports that he was on the phone recently talking to ‘his girl’”; “Pt was waiting to go to OR tonight with surgery for ‘my broken bones.’”	Stereotyping based on race, social class, and mechanism of injury was frequently noted. Rather than using objective medical terminology, HCPs used quotations to record unconventional grammar, culturally specific phrases, and communication with patients whose first language was not English, most notably when documenting reports of pain and medication requests. Records of patients with VP injuries consisted of significant injury bias, including insinuations around injury circumstances and speculative perceptions of safety risk. Violent injury was regularly and explicitly referred to as a barrier to arrangement of postdischarge services, including inpatient rehabilitation placement.
Difficult patient	Language that frames patients as uncooperative, unreasonable, argumentative, nonadherent, temperamental, or refusing or displays frustration with patients	“Though I try multiple times to engage with him, he says, ‘please...trying to get some rest.’”; “Patient is very needy and very difficult to engage in his own care”	“Continues to act belligerent and is verbally abusive to staff and yelling out. Pt demanding water though NPO for OR.”; “Pt and family very needy mother coming out frequently for help or knocking on the window.”	Difficult patient was the most common and consistent theme across the sample. Notes indicated HCPs’ perceiving patients as uncooperative, unreasonable, and argumentative. While records contained objective evidence that patients were experiencing trauma and pain, HCPs frequently overlooked or dismissed this in notes, attributing their challenges in administering care to patients’ intentional nonadherence. Overlapping with the disapproval theme, HCPs indicated discontent when patients required repetition in clinical instruction. Some notes focused on patients’ visitors, detailing challenging emotional behaviors, interference with care, and disruptive dynamics without offering more insight. The theme presented in similar fashion for patients with VC or MVC injuries.
Unilateral decisions	Displays an HCP-patient power dynamic through paternalistic language, lecturing, failing to consult patient on treatment course, presumption, or implying the patient is ignorant or childlike	“Again reminded patient if he actually wants to go home he needs to start doing for himself and not have his parents do it as he just becomes more deconditioned.”; “pt was not accepted to [rehabilitation center name] due to statement made by [city police department] that shooting may have been gang related”	“Pt expresses desire to have diet advanced and this writer informed her that her care team would make the decision of when to advance her diet.”	Notes demonstrated health care staff or law enforcement exercising authority over the patient, often limiting a patient’s agency through use of patronizing language. HCPs reported informing patients of their treatment plan with minimal or no documentation of attempts to provide explanation. Similarly, notes showed prioritization of the established care plan without considering adaptations based on patient-centered circumstances, intensifying power dynamics and corresponding with the disapproval and difficult patient theme. Authoritarian conditions regarding a patient’s disposition were exclusive to patients with VP injuries and often involved law enforcement, which extended decisions impacting medical care to individuals outside of health care and raised concern for injury bias and stereotyping. For patients with VP injuries, HCPs frequently attributed patients’ limited postdischarge options to their designated injury type.

Abbreviations: dtr, daughter; ETOH, alcohol; HCP, health care practitioner; MVC, motor vehicle crash; NPO, nil per os (nothing by mouth); OR, operating room; Pt, patient; VP, violent penetrating; yo, year-old.

^a^ Categories were derived and definitions were adapted from Park et al.^24^ Categories are not mutually exclusive and sometimes overlap.

## Discussion

This mixed-methods qualitative study of patients with trauma injuries discharged to rehabilitation centers found that, compared with patients with MVC injuries, patients with VP injuries faced more than triple the odds of being denied admission to a rehabilitation center. In most instances, the reasons for these denials were either not specified or patients were denied admission due to purported safety concerns. In our review of the medical records of patients in our study population, we frequently identified negative, stigmatizing language in the records of patients with VP injuries. Compared with patients with MVC injuries, HCPs’ notes included increased skepticism toward symptoms reported by patients with VP injuries, and these patients were often stereotyped as safety risks. Language reflecting power and authority over patients’ hospital discharge planning was exclusive to the records of patients with VP injuries. Together, these findings suggest the existence of implicit and explicit bias among HCPs toward patients with VP injuries, which can impact clinical decisions and outcomes and be transmitted among HCPs through documentation,^26,37,38,39^ likely harming patients’ self-identities, trust in HCPs, and treatment participation.^40,41^ To our knowledge, no published work has previously examined these dynamics.

Addressing inequitable access to rehabilitation services, particularly for patients with VP injuries, is an important part of population health. It is widely recognized that early engagement in comprehensive rehabilitation maximizes outcomes,^42^ including improved motor and cognitive function, increased independence, fewer subsequent emergency department visits, and decreased 1-year mortality.^4,5,28^ Participation in high-quality rehabilitation provides survivors of violence with unique access to services and resources, as many return to disadvantaged communities with barriers to follow-up care.^43,44^ Younger survivors of violence may face many years of disability and social marginalization if their rehabilitation needs are not met.^45^ The administrative barriers noted in our study align with prior literature indicating that most unnecessary hospital days among patients were caused by nonmedical factors, like issues with discharge planning,^11,46^ delaying patients’ recovery and straining organizational operations.^47^

Our main finding, that patients with VP injuries were at more than 3 times the odds of rehabilitation facility denial, also represents important issues of racial and health equity. Prior studies have established disparities in hospital discharge disposition among Black and Hispanic populations^6^ and survivors of violence, who are more likely to discharge home without services.^28^ In our study population of patients with traumatic injuries treated at BMC, 84% of patients with VP injuries identified as members of racial or ethnic minority groups, compared with 38% of patients with MVC injuries. In other words, discrimination against survivors of violence effectively constitutes discrimination against racially and ethnically minoritized individuals. In our univariate models, Black race was associated with greater odds of rehabilitation denial. This association was attenuated in the full model when other variables, including injury type and involvement of hospital public safety, were included. One possible interpretation is that among survivors of traumatic injuries, race is so inextricably linked with other factors, such as injury type, that its independent associations cannot be observed in a modestly sized sample. This linkage is due to structural racism.^48^ Furthermore, denials often result in patients being placed in less desired facilities and locations, leading to isolation and strain on social supports, particularly for those with limited socioeconomic resources.^8,12,49^

Bias and stigma embedded within the referral process likely contribute to the observed disparities in denials for admission to rehabilitation centers. In our study, both patients with VP injuries and patients with MVC injuries had documented instances of stigmatizing language in HCP notes, but records for patients with VP injuries consisted of explicit injury bias, overt doubt markers, and heightened HCP-patient power dynamics. While this is the first study, to our knowledge, exploring differences in stigmatizing language in electronic health records by injury type, prior studies have identified diagnosis-specific stigmatizing language related to diabetes, substance use, chronic pain, and obesity,^25,37^ as well as rhetoric indicating bias and judgment toward patients who are Black,^34,50^ Hispanic, women, or older than 65 years.^37^ Differences in documentation practices and implicit biases have also been noted by HCPs’ personal identities, title, and professional experience.^25,27^ This stigmatizing language may directly impact the decision-making of other HCPs on a patient’s care team and rehabilitation center representatives,^26,37,39^ potentially affecting patient outcomes through rehabilitation denials. Our analysis supports the standardization of documentation and referral practices for rehabilitation placement to reduce subjectivity and variability.

In many records for patients with VP injuries, CMs documented rehabilitation centers’ perfunctory requests to speak to law enforcement about the patient and circumstances surrounding their injury as part of their screening process, which sometimes contributed to delays in placement. When attempting to obtain a law enforcement contact, CMs’ documentation reflected the power and authority demonstrated by many rehabilitation centers over survivors of violence and their families. Facilities commonly referred to this step with survivors of violence as necessary for “security clearance” and to evaluate “safety risk.” No patients with MVC injuries were denied due to centers’ contact with police, whereas 14.5% of violence survivors had at least 1 denial due to “safety concerns.” These denials lacked formal documentation of the screening process and placed inappropriate discretion in the hands of law enforcement with limited potential for oversight. These practices demonstrate the integration of carceral systems into health care, which reinforces cycles of disempowerment and marginalization for survivors of violence. The involvement of law enforcement raises important questions about the informal processes occurring during referrals and whether patients’ HIPAA rights are being violated, and it further reflects prejudices held against patients with injuries caused by violence. The communication and documentation practices related to these exchanges are unknown. Treating violence survivors as threatening creates concerns for internalization of stigma and bias, which can influence patients’ desire to engage with health care and other essential systems.^51^

Our findings offer clear targets for reform efforts through policies and initiatives at the institutional and state levels. First, stigmatizing language in medical records should be addressed through ongoing efforts to develop and implement best practices for clinical communication, including standardized trauma-informed care training, implicit bias reduction strategies for HCPs, and evolved guidelines for language in documentation.^33,38,41,52^ We recommend standardization of these elements and that they include oversight with accountability measures. We found the rehabilitation referral process to be inconsistent, subjective, enigmatic, and poorly documented. While each level of rehabilitation has clear and strict regulations related to patients’ medical needs and functional status, patients, HCPs, and insurers consistently disagree on the suitability of placements. Establishing documentation and reporting requirements for referral screenings would enable greater transparency about these determinations. This would support the establishment of clear, measurable, and evidence-based discharge planning that could promote equitable access to care, departing from discretionary and seemingly biased practices. Improved systems for tracking long-term outcomes of trauma survivors are recommended, which could help with evaluation of patients’ posthospital placement and inform future discharge planning.^53^ Further research investigating the role and weight of specific factors during discharge planning are needed. Additionally, future studies should examine the perceptions of survivors of violence of the discharge planning process, their experiences receiving inpatient rehabilitation, their postrehabilitation quality of life, facilities’ quality metrics, and the greater societal costs of these disparities.

### Limitations

The results of this study are subject to limitations, including those related to study design and data constraints. First, our population included a modestly sized sample of patients with traumatic injuries treated at a single urban medical center and discharged to a rehabilitation center, which limits the generalizability of our findings to other patient populations at other hospitals with varying rehabilitation center availability. Second, we only evaluated patients with traumatic injuries in this analysis who were ultimately placed at an inpatient rehabilitation center after hospitalization. It is likely that numerous additional patients with traumatic injuries experienced barriers with referrals to inpatient rehabilitation and ultimately received a downgrade in discharge recommendation to home, especially considering that administrative delays and disposition disagreements among HCPs can contribute to patients abandoning treatment,^54^ and survivors of violence are more likely to have a downgrade in discharge recommendation for level of care compared with other patients with traumatic injuries.^20^ Third, disparate recording practices among health care practitioners and a lack of guidelines for CMs documenting the rehabilitation center referral process could contribute to incomplete information on reasons for rehabilitation center denials. Fourth, we were not able to determine whether a patient’s history of violent behavior within or outside of a clinical setting was associated with rehabilitation denial. While documentation of a patient’s history of violent behavior within the hospital can be found in the medical records, this is inconsistently documented, can be subjective or antiquated, and is itself subject to racial and gender bias,^55,56^ and we did not have access to criminal records. Furthermore, despite a robust literature search that informed our study, there may be unconsidered factors related to denials in this analysis due to the lack of transparency and standardized documentation requirements in the rehabilitation referral process.

## Conclusions

In this mixed-methods qualitative study of patients with traumatic injuries, we found that patients with violent injuries faced more than triple the odds of being denied admission to a posthospital inpatient rehabilitation center compared with patients with MVC injuries. Documentation in patients’ medical records contained multiple instances of negative, stigmatizing language. We observed that records of patients with VP injuries contained more overt instances of HCPs using stereotyping language, expressing skepticism of patients’ symptom reports, and practicing unilateral decision-making. These findings highlight a critical issue of racial and health equity in which policy changes and initiatives at the institutional and state levels could ensure equitable and ethical access to rehabilitation services for patients with traumatic injuries irrespective of the cause of their injury.

## Notes

Supplement 1. **eFigure.** Flowchart of Analysis **eTable 1.** Patient-Level Factors Associated With Any Facility Denial During the Discharge Process to Inpatient Rehabilitation Centers Following Admission to Boston Medical Center for Violent Penetrating or Motor Vehicle Crash Injuries, 2015-2021 **eTable 2.** Hospital Length of Stay by Number of Denials for Admission to Inpatient Rehabilitation Centers, 2015-2021 **eAppendix 1.** Quantitative Measures and Analysis **eAppendix 2.** Qualitative Data Collection and Analysis

Supplement 2.
Data Sharing Statement

## References

[1] Zhang S, Lin D, Wright ME, Swallow N. Acute inpatient rehabilitation improves function independent of comorbidities in medically complex patients. Arch Rehabil Res Clin Transl. 2022;4(2):100178. doi:10.1016/j.arrct.2022.100178 35756989 PMC9214302

[2] American College of Surgeons. National Trauma Data Bank 2016 annual report. Accessed February 26, 2025. https://www.facs.org/media/ez1hpdcu/ntdb-annual-report-2016.pdf

[3] Hall EC, Tyrrell R, Scalea TM, Stein DM. Trauma transitional care coordination: protecting the most vulnerable trauma patients from hospital readmission. Trauma Surg Acute Care Open. 2018;3(1):e000149. doi:10.1136/tsaco-2017-000149 29766133 PMC5887824

[4] Lancaster CW, DiMaggio C, Marshall G, Wall S, Ayoung-Chee P. Functional outcomes after inpatient rehabilitation for trauma-improved but unable to return home. J Surg Res. 2018;222:187-194.e3. doi:10.1016/j.jss.2017.09.024 29103674

[5] Nehra D, Nixon ZA, Lengenfelder C, . Acute rehabilitation after trauma: does it really matter? J Am Coll Surg. 2016;223(6):755-763. doi:10.1016/j.jamcollsurg.2016.09.001 28193321

[6] Haider AH, Weygandt PL, Bentley JM, . Disparities in trauma care and outcomes in the United States: a systematic review and meta-analysis. J Trauma Acute Care Surg. 2013;74(5):1195-1205. doi:10.1097/TA.0b013e31828c331d 23609267 PMC3641534

[7] Mayer RS, Noles A, Vinh D. Determination of postacute hospitalization level of care. Med Clin North Am. 2020;104(2):345-357. doi:10.1016/j.mcna.2019.10.011 32035573

[8] Qureshi AZ, Jenkins RM, Williamson LF. The broken link: admission criteria for inpatient rehabilitation and some common misconceptions. Saudi J Med Med Sci. 2016;4(3):233-237. doi:10.4103/1658-631X.188257 30787738 PMC6298344

[9] Centers for Medicare and Medicaid Services. Inpatient Rehabilitation Facility (IRF) Reference Booklet. Accessed February 26, 2025. https://www.cms.gov/files/document/inpatientrehabilitationfacilityrefbooklet2pdf

[10] Jaffe KM, Jimenez N. Disparity in rehabilitation: another inconvenient truth. Arch Phys Med Rehabil. 2015;96(8):1371-1374. doi:10.1016/j.apmr.2015.04.017 25958194 PMC4871110

[11] Buntin MB, Garten AD, Paddock S, Saliba D, Totten M, Escarce JJ. How much is postacute care use affected by its availability? Health Serv Res. 2005;40(2):413-434. doi:10.1111/j.1475-6773.2005.0i366.x 15762900 PMC1361149

[12] Levine C, Ramos-Callan K. The illusion of choice: why decisions about post-acute care are difficult for patients and family caregivers. *United Hospital Fund*. January 9, 2019. Accessed February 26, 2025. https://uhfnyc.org/publications/publication/patient-and-caregiver-perspectives-discharge-planning/

[13] Lussiez A, Montgomery JR, Sangji NF, . Hospital effects drive variation in access to inpatient rehabilitation after trauma. J Trauma Acute Care Surg. 2021;91(2):413-421. doi:10.1097/TA.0000000000003215 34108424 PMC8375412

[14] Meagher AD, Beadles CA, Doorey J, Charles AG. Racial and ethnic disparities in discharge to rehabilitation following traumatic brain injury. J Neurosurg. 2015;122(3):595-601. doi:10.3171/2014.10.JNS14187 25415069

[15] Englum BR, Villegas C, Bolorunduro O, . Racial, ethnic, and insurance status disparities in use of posthospitalization care after trauma. J Am Coll Surg. 2011;213(6):699-708. doi:10.1016/j.jamcollsurg.2011.08.017 21958511 PMC3684147

[16] Bowman SM, Martin DP, Sharar SR, Zimmerman FJ. Racial disparities in outcomes of persons with moderate to severe traumatic brain injury. Med Care. 2007;45(7):686-690. doi:10.1097/MLR.0b013e31803dcdf3 17571018

[17] Haider AH, Chang DC, Efron DT, Haut ER, Crandall M, Cornwell EE III. Race and insurance status as risk factors for trauma mortality. Arch Surg. 2008;143(10):945-949. doi:10.1001/archsurg.143.10.945 18936372

[18] Patton D, Sodhi A, Affinati S, Lee J, Crandall M. Post-Discharge needs of victims of gun violence in Chicago: a qualitative study. J Interpers Violence. 2019;34(1):135-155. doi:10.1177/0886260516669545 27638688

[19] Liebschutz J, Schwartz S, Hoyte J, . A chasm between injury and care: experiences of black male victims of violence. J Trauma. 2010;69(6):1372-1378. doi:10.1097/TA.0b013e3181e74fcf 20838259 PMC3005415

[20] Janeway MG, Cornell E, Smith SM, . Disparities in rehabilitation services for victims of violence. J Surg Res. 2025;306:317-326. doi:10.1016/j.jss.2024.12.040 39842045

[21] Nistler CM, James TL, Dugan E, Pino EC. Racial and ethnic disparities in violent penetrating injuries and long-term adverse outcomes. J Interpers Violence. 2023;38(3-4):2286-2312. doi:10.1177/08862605221101395 35604722

[22] Bazargan M, Cobb S, Assari S. Discrimination and medical mistrust in a racially and ethnically diverse sample of California adults. Ann Fam Med. 2021;19(1):4-15. doi:10.1370/afm.2632 33431385 PMC7800756

[23] Smith R, Dobbins S, Evans A, Balhotra K, Dicker RA. Hospital-based violence intervention: risk reduction resources that are essential for success. J Trauma Acute Care Surg. 2013;74(4):976-980. doi:10.1097/TA.0b013e31828586c9 23511134

[24] Park J, Saha S, Chee B, Taylor J, Beach MC. Physician use of stigmatizing language in patient medical records. JAMA Netw Open. 2021;4(7):e2117052. doi:10.1001/jamanetworkopen.2021.17052 34259849 PMC8281008

[25] Himmelstein G, Bates D, Zhou L. Examination of stigmatizing language in the electronic health record. JAMA Netw Open. 2022;5(1):e2144967. doi:10.1001/jamanetworkopen.2021.44967 35084481 PMC8796019

[26] P Goddu A, O’Conor KJ, Lanzkron S, . Do words matter: stigmatizing language and the transmission of bias in the medical record. J Gen Intern Med. 2018;33(5):685-691. doi:10.1007/s11606-017-4289-2 29374357 PMC5910343

[27] FitzGerald C, Hurst S. Implicit bias in healthcare professionals: a systematic review. BMC Med Ethics. 2017;18(1):19. doi:10.1186/s12910-017-0179-8 28249596 PMC5333436

[28] Gontarz BR, Siddiqui U, McGuiness C, . Victims of violence and post-discharge adverse events: a prospective modified Trauma Quality Improvement Program (TQIP) Study. Cureus. 2021;13(10):e18630. doi:10.7759/cureus.18630 34786230 PMC8580117

[29] Elo S, Kyngäs H. The qualitative content analysis process. J Adv Nurs. 2008;62(1):107-115. doi:10.1111/j.1365-2648.2007.04569.x 18352969

[30] Creswell JW. Designing and Conducting Mixed Methods Research and the Mixed Methods Research Workbook. SAGE Publications; 2021.

[31] Dawadi S, Shrestha S, Giri RA. Mixed-methods research: a discussion on its types, challenges, and criticisms. J Pract Stud Educ. 2021;2(2):25-36. doi:10.46809/jpse.v2i2.20

[32] Elfil M, Negida A. Sampling methods in clinical research: an educational review. Emerg (Tehran). 2017;5(1):e52. 28286859 PMC5325924

[33] Healy M, Richard A, Kidia K. How to reduce stigma and bias in clinical communication: a narrative review. J Gen Intern Med. 2022;37(10):2533-2540. doi:10.1007/s11606-022-07609-y 35524034 PMC9360372

[34] Sun M, Oliwa T, Peek ME, Tung EL. Negative patient descriptors: documenting racial bias in the electronic health record. Health Aff (Millwood). 2022;41(2):203-211. doi:10.1377/hlthaff.2021.01423 35044842 PMC8973827

[35] Pino EC, Fontin F, James TL, Dugan E. Boston Violence Intervention Advocacy Program: challenges and opportunities for client engagement and goal achievement. Acad Emerg Med. 2021;28(3):281-291. doi:10.1111/acem.14162 33111373

[36] Lumivero. NVivo. Accessed February 26, 2025. https://lumivero.com/

[37] Chapman EN, Kaatz A, Carnes M. Physicians and implicit bias: how doctors may unwittingly perpetuate health care disparities. J Gen Intern Med. 2013;28(11):1504-1510. doi:10.1007/s11606-013-2441-1 23576243 PMC3797360

[38] Zestcott CA, Blair IV, Stone J. Examining the presence, consequences, and reduction of implicit bias in health care: a narrative review. Group Process Intergroup Relat. 2016;19(4):528-542. doi:10.1177/1368430216642029 27547105 PMC4990077

[39] Barcelona V, Scharp D, Idnay BR, Moen H, Cato K, Topaz M. Identifying stigmatizing language in clinical documentation: A scoping review of emerging literature. PLoS One. 2024;19(6):e0303653. doi:10.1371/journal.pone.0303653 38941299 PMC11213326

[40] Entwistle VA, Carter SM, Cribb A, McCaffery K. Supporting patient autonomy: the importance of clinician-patient relationships. J Gen Intern Med. 2010;25(7):741-745. doi:10.1007/s11606-010-1292-2 20213206 PMC2881979

[41] Odero A, Pongy M, Chauvel L, . Core values that influence the patient-healthcare professional power dynamic: steering interaction towards partnership. Int J Environ Res Public Health. 2020;17(22):8458. doi:10.3390/ijerph17228458 33203162 PMC7696821

[42] Sirois MJ, Lavoie A, Dionne CE. Impact of transfer delays to rehabilitation in patients with severe trauma. Arch Phys Med Rehabil. 2004;85(2):184-191. doi:10.1016/j.apmr.2003.06.009 14966701

[43] Semenza DC, Stansfield R. Community gun violence and functional disability: an ecological analysis among men in four U.S. cities. Health Place. 2021;70:102625. doi:10.1016/j.healthplace.2021.102625 34280714

[44] Brandolino A, deRoon-Cassini TA, Biesboer EA, . Improved follow-up care for gun violence survivors in the Trauma Quality of Life Clinic. Trauma Surg Acute Care Open. 2024;9(1):e001199. doi:10.1136/tsaco-2023-001199 38390473 PMC10882323

[45] Ralph L. Renegade Dreams: Living Through Injury in Gangland Chicago. University of Chicago Press; 2014. doi:10.7208/chicago/9780226032856.001.0001

[46] Carey MR, Sheth H, Braithwaite RS. A prospective study of reasons for prolonged hospitalizations on a general medicine teaching service. J Gen Intern Med. 2005;20(2):108-115. doi:10.1111/j.1525-1497.2005.40269.x 15836542 PMC1490052

[47] Micallef A, Buttigieg SC, Tomaselli G, Garg L. Defining delayed discharges of inpatients and their impact in acute hospital care: a scoping review. Int J Health Policy Manag. 2022;11(2):103-111. 32610822 10.34172/ijhpm.2020.94 PMC9278600

[48] Jay J. Structural racism and long-term disparities in youth exposure to firearm violence. JAMA Netw Open. 2023;6(5):e2312425. doi:10.1001/jamanetworkopen.2023.12425 37159204 PMC10883295

[49] Gledhill K, Bucknall TK, Lannin NA, Hanna L. The role of collaborative decision-making in discharge planning: perspectives from patients, family members and health professionals. J Clin Nurs. 2023;32(19-20):7519-7529. doi:10.1111/jocn.16820 37403644

[50] Beach MC, Saha S, Park J, . Testimonial injustice: linguistic bias in the medical records of Black patients and women. J Gen Intern Med. 2021;36(6):1708-1714. doi:10.1007/s11606-021-06682-z 33754318 PMC8175470

[51] Drapalski AL, Lucksted A, Perrin PB, . A model of internalized stigma and its effects on people with mental illness. Psychiatr Serv. 2013;64(3):264-269. doi:10.1176/appi.ps.001322012 23573532

[52] Wathen CN, MacGregor JCD, Beyrem S. Impacts of trauma- and violence-informed care education: a mixed method follow-up evaluation with health & social service professionals. Public Health Nurs. 2021;38(4):645-654. doi:10.1111/phn.12883 33629448

[53] Haider AH, Herrera-Escobar JP, Al Rafai SS, . Factors associated with long-term outcomes after injury: results of the Functional Outcomes and Recovery After Trauma Emergencies (FORTE) multicenter cohort study. Ann Surg. 2020;271(6):1165-1173. doi:10.1097/SLA.0000000000003101 30550382

[54] American Medical Association. 2023 AMA prior authorization physician survey. Accessed February 26, 2025. https://www.ama-assn.org/system/files/prior-authorization-survey.pdf

[55] Agarwal AK, Seeburger E, O’Neill G, . Prevalence of behavioral flags in the electronic health record among Black and White patients visiting the emergency department. JAMA Netw Open. 2023;6(1):e2251734. doi:10.1001/jamanetworkopen.2022.51734 36656576 PMC9857105

[56] Haimovich AD, Taylor RA, Chang-Sing E, . Disparities associated with electronic behavioral alerts for safety and violence concerns in the emergency department. Ann Emerg Med. 2024;83(2):100-107. doi:10.1016/j.annemergmed.2023.04.004 37269262 PMC10689576

