# Supplementary material for: Health Care Practitioner Bias and Access to Inpatient Rehabilitation Services Among Survivors of Violence
Source: JAMA Netw Open. 2025 Apr 8;8(4):e254074. doi: 10.1001/jamanetworkopen.2025.4074 (PMC11979725; doi:10.1001/jamanetworkopen.2025.4074)
Supplement: Supplement 1. — eFigure. Flowchart of Analysis eTable 1. Patient-Level Factors Associated With Any Facility Denial During the Discharge Process to Inpatient Rehabilitation Centers Following Admission to Boston Medical Center for Violent Penetrating or Motor Vehicle Crash Injuries, 2015-2021 eTable 2. Hospital Length of Stay by Number of Denials for Admission to Inpatient Rehabilitation Centers, 2015-2021 eAppendix 1. Quantitative Measures and Analysis eAppendix 2. Qualitative Data Collection and Analysis [file jamanetwopen-e254074-s001.pdf]

Supplemental Online Content

Georges MR, Courtepatte A, Hibara A, et al. Health care practitioner bias and access to inpatient rehabilitation services among survivors of violence. *JAMA Netw Open*. 2025;8(4):e254074. doi:10.1001/jamanetworkopen.2025.4074

eFigure. Flowchart of Analysis

eTable 1. Patient-Level Factors Associated With Any Facility Denial During the Discharge Process to Inpatient Rehabilitation Centers Following Admission to Boston Medical Center for Violent Penetrating or Motor Vehicle Crash Injuries, 2015-2021

eTable 2. Hospital Length of Stay by Number of Denials for Admission to Inpatient Rehabilitation Centers, 2015-2021

eAppendix 1. Quantitative Measures and Analysis

eAppendix 2. Qualitative Data Collection and Analysis

This supplemental material has been provided by the authors to give readers additional information about their work.

© 2025 Georges MR et al. *JAMA Network Open*.

eFigure. Flowchart of Analysis

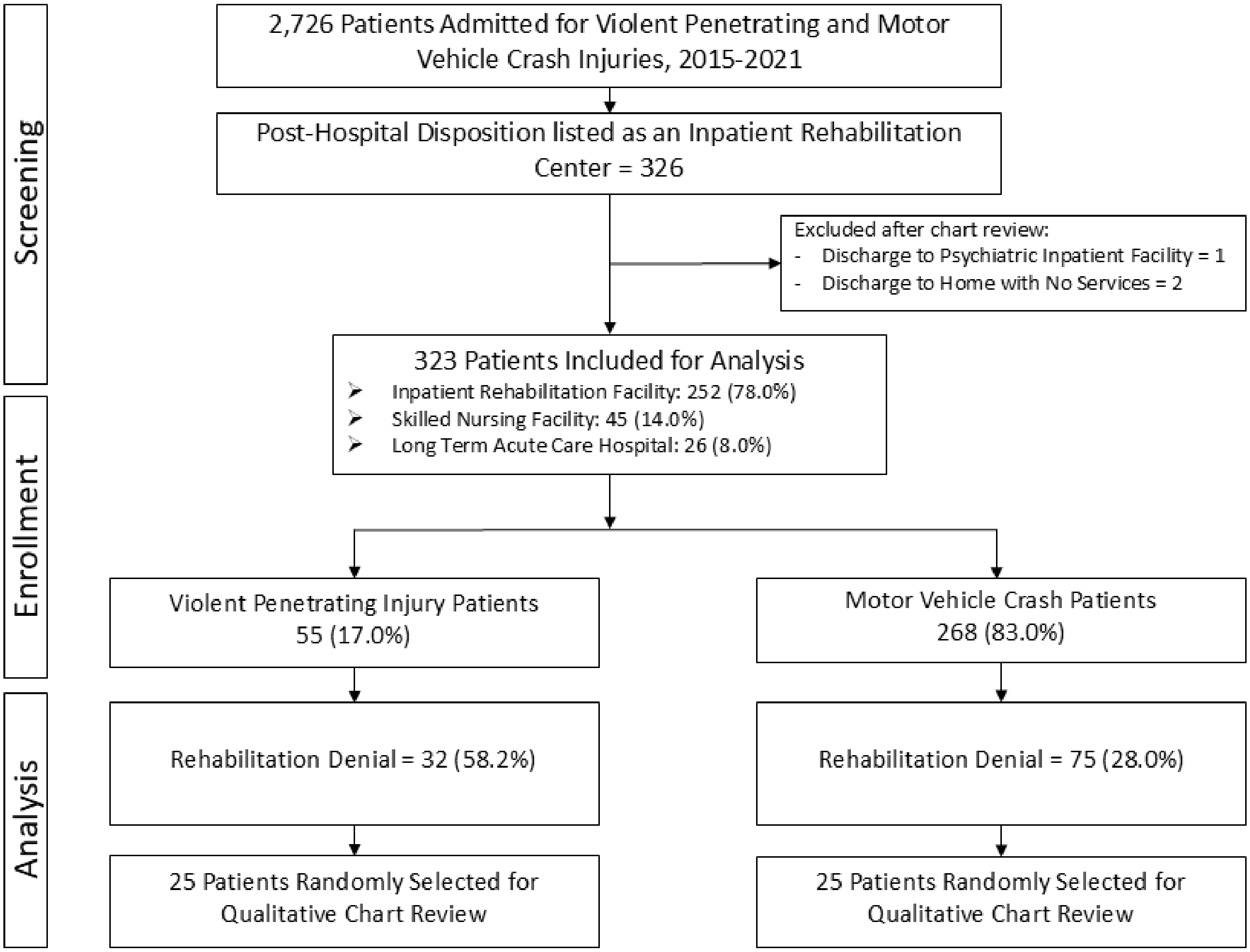

© 2025 Georges MR et al. *JAMA Network Open*.

eTable 1: Patient-level factors associated with any facility denial during the discharge process to inpatient rehabilitation centers following admission to Boston Medical Center for violent penetrating or motor vehicle crash injuries, 2015 – 2021

	Patients Denied[a]	Odds of Rehabilitation Denial for Any Reason[b]					
		Crude		Full Multivariable Model		Final Multivariable Model	
	n (%)	OR (95% CI)	*p*	OR (95% CI)	*p*	OR (95% CI)	*P*
**Total Patients Denied**	107 (33.1)						
Injury Type			<0.0001		0.18		<0.0001
Motor Vehicle Crash	75 (28.0)	Ref		Ref		Ref	
Violent Penetrating	32 (58.2)	3.58 (1.98, 6.58)		1.93 (0.73, 5.09)		3.51 (1.93, 6.48)	
Age		0.99 (0.98, 1.00)	0.23	1.00 (0.99, 1.02)	0.63		
Injury Year		0.98 (0.87, 1.09)	0.67	0.98 (0.87, 1.11)	0.77		
Gender			0.44		0.92		
Female	35 (30.4)	Ref		Ref			
Male	72 (34.6)	1.21 (0.75, 1.99)		0.97 (0.58, 1.65)			
Race and Ethnicity			0.05		0.57		
Non-Hispanic White	39 (25.7)	Ref		Ref			
Non-Hispanic Black	49 (41.5)	2.06 (1.23, 3.46)		1.53 (0.83, 2.82)			
Hispanic (any race)	10 (34.5)	1.52 (0.63, 3.51)		1.10 (0.41, 2.80)			
Other or Unknown[c]	9 (37.5)	1.74 (0.68, 4.23)		1.27 (0.46, 3.29)			
Housing Barrier			0.03		0.66		
No	99 (31.9)	Ref		Ref			
Yes	8 (61.5)	3.41 (1.11, 11.53)		1.33 (0.37, 5.12)			
Mental Health Diagnosis			0.08		0.13		
No	75 (30.5)	Ref		Ref			
Yes	32 (41.6)	1.62 (0.95, 2.75)		1.51 (0.87, 2.88)			
Hospital Public Safety Called			0.02		0.08		0.04
No	96 (31.6)	Ref		Ref		Ref	
Yes	11 (57.9)	2.98 (1.17, 7.92)		2.47 (0.89, 7.05)		2.81 (1.07, 7.66)	
Police Contact			0.0001		0.21		
No	79 (28.6)	Ref		Ref			
Yes	28 (59.6)	3.67 (1.95, 7.05)		1.87 (0.69, 5.04)			

[a] Values in first column represent frequencies and row percentages.

© 2025 Georges MR et al. *JAMA Network Open*.

[b] Logistic regression models were used for estimating odds ratio (OR), 95% confidence intervals (95%CI) and *p* values. Multivariable model is adjusted for injury type and hospital public safety called.

[c] "Other or Unknown" Race and Ethnicity includes patients who were Asian, American Indian or Alaskan Native, or patients who chose not to respond when asked about their self-identified race and ethnicity.

© 2025 Georges MR et al. *JAMA Network Open*.

eTable 2: Hospital length of stay by number of denials for admission to inpatient rehabilitation centers, 2015 – 2021.

No. of denials	No. of patients, (%)	Median (IQR) LOS, days
**0**	216 (66.9)	9.0 (5.5 – 12.5)
**1**	61 (18.9)	12.0 (4.0 – 20.0)
**≥2**	46 (14.2)	20.3 (9.9 – 30.6)

© 2025 Georges MR et al. *JAMA Network Open*.

eAppendix 1. Quantitative Measures and Analysis

Patient demographics were abstracted from the medical record. Age was measured as a continuous variable at the time of injury. Gender was categorized as male or female based on patient's self-reported gender. We created four categories from patients' self-reported race and ethnicity, which is collected by hospital registration staff during a patient's visit: non-Hispanic White, Hispanic (any race), non-Hispanic Black, and non-Hispanic Other/Unknown (includes Asian, American Indian or Alaskan Native, and patients who chose not to respond). Hospital length of stay was measured in days as continuous.

Additional binary variables determined by chart review and clinical background to have potential relevance to the rehabilitation referral process were housing barrier, mental health diagnosis, hospital public safety utilization, and facility follow-up with law enforcement. Patients were considered to have a housing barrier if the patient's medical chart during their hospitalization included a provider's note explicitly stating that the patient did not have stable housing to discharge to from a rehabilitation center. Mental health diagnoses included those listed in patients' past medical history or explicitly noted by a clinician in their charts, which included anxiety, depression, attention-deficit/hyperactivity disorder, bipolar disorder, post-traumatic stress disorder, schizophrenia, or borderline personality disorder. Hospital public safety called indicates that it was documented in a patient's chart that public safety was paged by hospital providers for assistance with either the patient or associated visitors, including remaining on standby, escorting visitors out of the hospital, and assisting with the application of violent restraints to a patient. Police contact indicates that a patient's chart noted that a rehabilitation center required being provided the contact information of law enforcement assigned to any criminal investigation related to the events that resulted in a patient's injuries in order to complete admission screening.

Patient characteristics were compared by injury type: violent compared to MVC injury. Continuous variables were summarized with medians and interquartile ranges (IQR), with group comparisons conducted using the Wilcoxon rank sum test, after rejection of the assumption of normality using the Shapiro-Wilks test. Categorical variables were summarized with frequencies and percentages, with group comparisons conducted using Chi-square tests or Fisher's exact test. Variables with missing information (unknown) were included in the analysis, and data imputation was not used to replace missing values.

Logistic regression models were used to calculate odds ratios (OR) and 95% confidence intervals (95% CI) for associations between patient injury type and rehabilitation center denials. No violations of collinearity among predictor variables were observed. Crude univariate estimates were derived for each covariate. The full multivariable models included all covariates listed in table 1. The final models were constructed using stepwise selection, and statistical significance was considered to be P<.05. The final multivariable logistic regression model for rehabilitation center denial was adjusted for injury type and utilization of hospital public safety. Model goodness of fit was evaluated using the Hosmer-Lemeshow test. We reported logistic regression results as odds ratios (ORs) with 95% confidence intervals (CI). Data analysis was conducted using R.[32]

© 2025 Georges MR et al. *JAMA Network Open*.

eAppendix 2. Qualitative Data Collection and Analysis

First, three coders independently tested a draft codebook by reading through the same two randomly selected patient charts – one for each injury type. These charts were excluded when selecting the final sample for the qualitative portion. Coders took notes throughout the coding process and inductively expanded category definitions for clarity as needed, leveraging additional studies to strengthen category definitions and identify common words and syntax that suggest stigma.[25, 34, 37] Coders then met to discuss testing to achieve consensus and finalize the codebook.

Three researchers, two masters-level public health researchers (A.C. and A.H.) and a masters-level data analyst with training in text analysis (M.R.G.), independently analyzed the data using NVivo Release 1.7.1 (QSR International).[38] We evenly split the 25 violence survivor and 25 MVC patient charts among the three coders. Each coder independently reviewed their assigned charts, copying sections of chart notes containing stigmatizing language and surrounding context into their individual NVivo file and assigning codes. We used a 'help' code when uncertain about specific entries, which were discussed as a group after finishing all chart reviews to deliberate and reach consensus.

Coders' NVivo files were then consolidated to one master file, and the three coders performed qualitative content analysis[29] to explore the presence of stigmatizing language in patients' charts and compare the coded material for the categories by injury type. Using NVivo's query tool, we grouped all abstractions by stigmatizing category code and injury type. Each category's coded material was randomly assigned to a coder to review independently and draft category summaries, focusing on differences by injury type. The drafts were then swapped among coders and reviewed for accuracy and completeness. Summaries were reviewed by an emergency medicine physician for clinical accuracy and to check for inappropriate extrapolations.

© 2025 Georges MR et al. *JAMA Network Open*.
