# Supplementary material for: Health Care Practitioner Bias and Access to Inpatient Rehabilitation Services Among Survivors of Violence
Source: JAMA Netw Open. 2025 Apr 8;8(4):e254074. doi: 10.1001/jamanetworkopen.2025.4074 (PMC11979725; doi:10.1001/jamanetworkopen.2025.4074)
Supplement: Supplement 2. — Data Sharing Statement [file jamanetwopen-e254074-s002.pdf]

Data Sharing Statement

Georges. Health Care Practitioner Bias and Access to Inpatient Rehabilitation Services Among Survivors of Violence. *JAMA Netw Open*. Published April 08, 2025. doi:10.1001/jamanetworkopen.2025.4074

Data
Data available: No
